# The indicative effects of apolipoproteins on organic erectile dysfunction: bridging Mendelian randomization and case-control study

**DOI:** 10.3389/fendo.2024.1359015

**Published:** 2024-06-13

**Authors:** Zhexin Zhang, Mo Yan, Yuezheng Li, Yang Pan, Shangren Wang, Mingming Xu, Hang Zhou, Xiaoqiang Liu

**Affiliations:** ^1^ Department of Urology, Xiangya Hospital, Central South University, Changsha, China; ^2^ Department of Urology, Tianjin Medical University General Hospital, Tianjin, China

**Keywords:** apolipoprotein, Mendelian randomization, diagnosis, erectile dysfunction, the nocturnal penile tumescence and rigidity test

## Abstract

The existing research on the association between apolipoproteins (Apos) and erectile dysfunction (ED) primarily relies on observational studies and does not distinguish between organic and psychogenic causes when diagnosing ED. It is difficult to believe that Apos play a role in psychogenic ED. To address these issues, our study explored the causal relationship between lipoproteins and ED using Mendelian randomization (MR) analysis and differentiate between organic and psychogenic ED through the use of nocturnal penile tumescence and rigidity (NPTR) monitoring. Multivariate MR analysis revealed significant causal associations between high-density lipoprotein (HDL), Apo A1, and Apo B/A1 with ED (OR and 95% CI were 0.33 (0.14-0.78), 3.58 (1.52-8.43), and 0.30 (0.13-0.66)). we conducted statistical and analytical analyses on the data of 212 patients using multivariate analyses and receiver operating characteristic (ROC) curves. Patients with organic ED had significantly lower levels of HDL, Apo A1 and Apo A1/B, whereas patients with organic ED had considerably higher levels of Apo B and low-density lipoprotein (LDL). The diagnostic value of Apos in predicting the risk of organic ED was evaluated using ROC curves. The results indicated that Apo A1 and Apo A1/B demonstrated good predictive value. HDL, Apo A1, and Apo A1/B have been identified as risk factors for ED in our study. Furthermore, our research highlights the significance of Apo A1 and Apo A1/Apo B in the development of organic ED and suggests their potential use as indicators to assess the risks associated with organic ED.

## Introduction

Erectile dysfunction (ED) denotes the persistent incapacity to attain and/or sustain a sufficiently robust erection to achieve satisfactory sexual performance (1). Approximately 10-25% of men are affected by ED, a prevalent sexual condition worldwide (2). It is expected that the frequency and prevalence rates of ED will rise. It is projected that around 322 million individuals will be diagnosed with ED by 2025 (3).

Numerous studies have demonstrated a shared pathophysiological basis of ED and cardiovascular diseases (CVDs) in terms of vascular insufficiency (4). Dyslipidemia, a disorder of lipoprotein metabolism, is widely recognized as the primary risk factor for CVD. Moreover, clinicians have utilized lipoproteins such as low-density lipoprotein (LDL), high-density lipoprotein (HDL), total cholesterol (TC), triglycerides (TG), and apolipoproteins (Apos) to predict ED due to the close pathophysiological relationship between CVD and ED (5, 6). Notably, Apos like Apo B, Apo A1, and Apo A1/B have proven to be highly effective in predicting CVD in the general population, as they indicate vascular endothelium damage (7, 8). A previous study has suggested that Apo B, Apo A1, and Apo A1/Apo B can also serve as risk factors for ED (9). However, determining the respective contribution of each factor to ED is challenging due to the complex interrelationships among these symptoms, even though many cases of ED can be attributed to a combination of dyslipidemia risk factors.

ED is a common male sexual dysfunction that can be caused by various factors, such as vascular, neurogenic, hormonal, and anatomic abnormalities, as well as psychological and mixed factors (10). The International Index of Erectile Function (IIEF-5), a widely used multidimensional tool, is valuable for evaluating male sexual function and serves as a useful resource for examining ED. It is recommended to utilize this tool as a starting point for ED testing and to assess the severity of ED (11). Additionally, nocturnal penile tumescence and rigidity (NPTR) occurs during rapid eye movement sleep independently of sexual stimulation, resulting in sleep-related erections. This concept is based on the idea that if a man with ED experiences normal nocturnal erectile activity, the cause may be ‘psychogenic’ (12). Psychogenic factors such as stress, anxiety, distress, depression, and relationship problems do not typically affect sleep-related erections. On the other hand, men with normal erectile function usually experience 4-6 episodes of involuntary nocturnal erections lasting 20-50 minutes within a 6-8 hour sleep cycle (13). NPTR monitoring was the first objective test used for diagnosing ED and has been considered a useful and reliable method for distinguishing between psychogenic and organic ED (14).

Despite the numerous assertions made, a significant portion of the research relied on observation. However, it is important to note that observational studies are limited in their ability to establish causality. Therefore, it is necessary to employ other research methods to minimize the influence of confounding factors. One such method is Mendelian randomization (MR) analysis, which offers distinct advantages over observational studies. MR analysis effectively addresses confounding and reverse causality by utilizing instrumental variables (IVs) in the form of single nucleotide polymorphisms (SNPs), rather than genetic variation. This approach is particularly valuable when examining risk factors that may be prone to measurement inaccuracies (15). Additionally, the introduction of multivariable MR (MVMR) as a new MR model allows for the direct evaluation of each risk factor’s individual impact, thereby mitigating the potential influence of other related risk factors (16, 17).

The primary objective of this study is to utilize both single variable MR (SVMR) and MVMR to assess the overall and immediate impacts of lipid characteristics on the risk of ED initially. Previous studies have used the IIEF-5 diagnostic modality for ED and the genome-wide association study (GWAS) database. However, the IIEF-5 diagnosis encompasses both organic and psychogenic causes of ED, and it is difficult to believe that Apos play a role in psychogenic ED. Therefore, we employed NPTR monitoring for ED diagnosis, which provides a more objective means to distinguish between organic and psychogenic ED. This helps us verify the correlation between Apos and organic ED, excluding psychogenic ED.

## Materials and methods

### MR design

**Figure 1:**
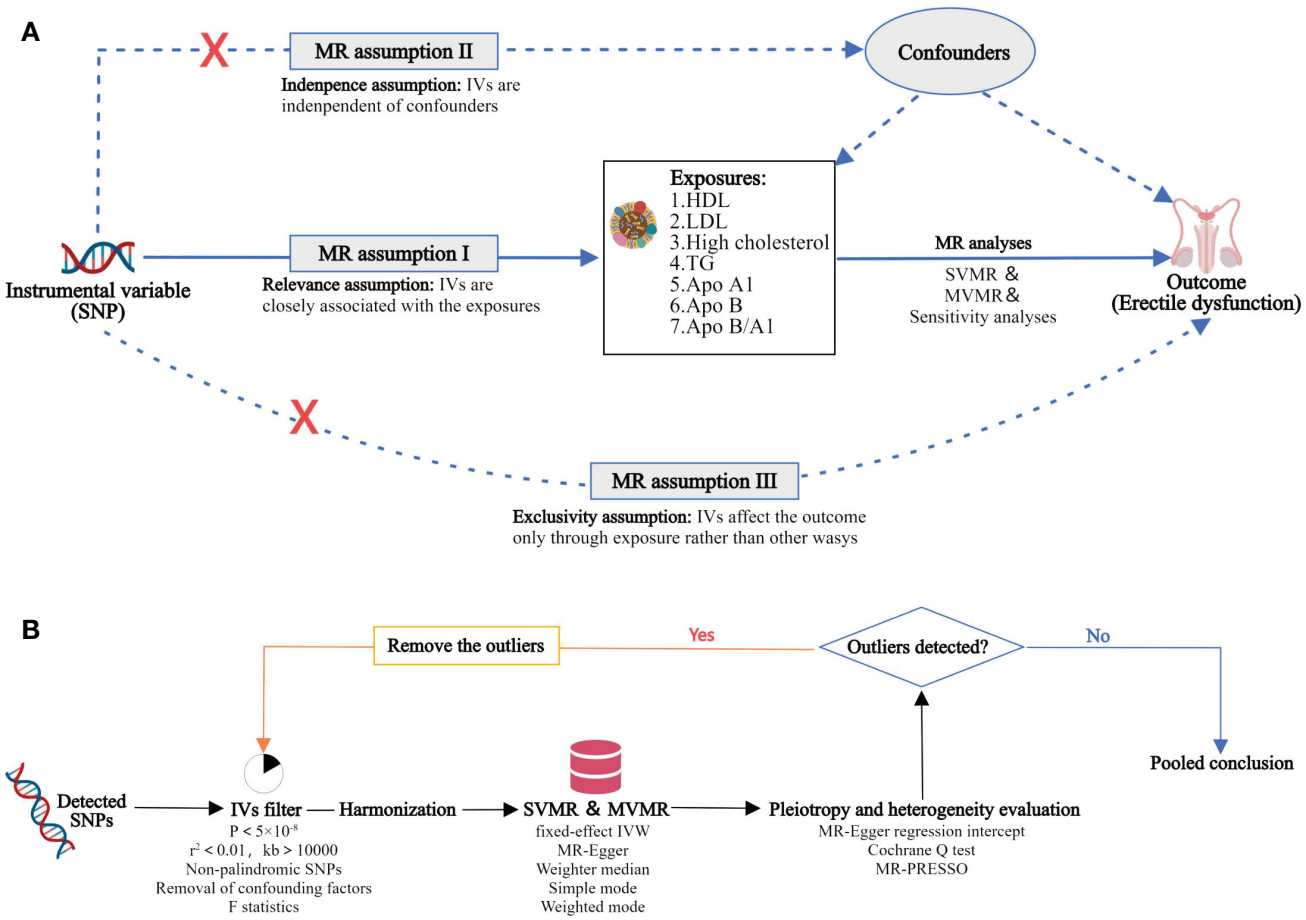
Overview of the MR study design and analysis strategy. **(A)**: Study design overview: The study examines the effects of 7 lipoproteins, namely HDL, LDL, high cholesterol, TG, Apo A1, Apo B, and Apo B/A1. The MR framework is based on three fundamental MR assumptions. **(B)**: MR analysis strategy: Initially, qualified SNPs are filtered as IVs. Subsequently, sensitivity analyses are conducted to assess the robustness of the results, along with the evaluation of heterogeneity and pleiotropy. MR, Mendelian randomization; SNP, single nucleotide polymorphism; IV, instrumental variable; HDL, high-density lipoprotein; LDL, low-density lipoprotein; TG, triglycerides; Apo, apolipoprotein; SVMR, single variable MR; MVMR, multivariate MR; MR‐PRESSO, MR Pleiotropy RESidualSum and Outlier.

MR has three primary conjectures. Hypothesis 1 suggests a strong correlation between genetic variants used as independent variables and traits associated with lipids. Hypothesis 2 states that the independent variables used should not be connected to any potential confounding variables. Hypothesis 3 proposes that the risk of the outcome should only be influenced by exposed genetic variants through risk factors, rather than through alternative pathways. In order to investigate the potential cause-and-effect relationship between lipoproteins and ED, we conducted a MR study using the logic of two-sample MR. The purpose of the SVMR analysis was to examine the correlation between individual lipoproteins and ED outcomes, while the MVMR analysis aimed to compare the independent role of lipoprotein-associated traits in ED outcomes. The study design of this experiment is illustrated in **Figure 1**.

### Sample source

SNPs identified by large samples of GWASs were used as IVs to represent exposures of lipoproteins, including LDL, HDL, high cholesterol, TG, and Apos. **Table 1** presents a comprehensive breakdown of the sample information in this study.

**Table 1:** Summary of genome-wide association studies included in this study.

Phenotype	GWAS data source	Cohort (s)	Sample size	Race
Apo A1	(18)	UK Biobank	115082	European
Apo B	(18)	UK Biobank	115082	European
Apo B/A1	(18)	UK Biobank	115082	European
HDL	(19)	UK Biobank	357810	European
LDL	(19)	UK Biobank	389189	European
High cholesterol	(20)	UK Biobank	484598	European
TG	(21)	UK Biobank	441016	European
ED	FinnGen	FinnGen	95178	European

Apo, apolipoprotein; HDL, high-density lipoprotein; LDL, low-density lipoprotein; TG, triglycerides; ED, erectile dysfunction.

We made use of the Apo A1, ApoB, and Apo B/A data sourced from Tom G Richardson et al. After analyzing the information from The UK Biobank, a conclusion was reached (18). HDL, LDL, high cholesterol and TG data were obtained from The UK Biobank (http://www.ukbiobank.ac.uk/, accessed on 18 October 2023) (19–21).

In this study, the GWAS dataset of ED from FinnGen (https://r7.finngen.fi/, accessed on 20 October 2023) were used as outcome datasets. The study included a total of 1154 cases and 94024 controls, all of whom were men from European Finland. ED was identified based on the International Classification of Diseases, 10th Revision (ICD-10) codes (N48.4 and F52.2), or through medical records of ED intervention, such as surgical procedures or oral medication, or self-reported information provided by the participants.

### Selection of IVs

To ensure statistical power, we selected SNPs with significant associations with lipoproteins (p < 5 × 10^−8^) as independent variables, as shown in **Figure 1**. We then excluded chain disequilibrium responses by applying thresholds of r^2^ < 0.001 and Kb > 10,000. Subsequently, we retrieved the effect estimates of the selected independent variables from the ‘ED outcome’ dataset. To address the second hypothesis of MR, we utilized the PhenoScannerV2 database (http://www.phenoscanner.medschl.cam.ac.uk/), eliminating SNPs associated with ED outcomes and confounding variables (22). Earlier studies have indicated that fasting glucose, uric acid, and hypertension could potentially contribute to the development of ED (23–25). To ensure the accuracy of our study, we excluded fasting glucose, uric acid, and hypertension as potential confounding factors. We also avoided using palindromic SNPs, which are characterized by having the same nucleotides paired together in a DNA molecule (26). In order to meet the Mendelian first hypothesis, we used R2 as a genetic tool to explain the proportion of trait variance. R^2^ was calculated using the formula R^2 = ^2 × (1 − MAF) × MAF × β^2^/(SE^2^ × N), where N represents the sample size, β is the effect size, SE is the standard error, and MAF is the minimum allele frequency for each SNP. Additionally, we computed an F statistic to assess the overall potency of the selected SNPs in explaining phenotypic variability. The equation used for this calculation was F = β²/SE². A value of F greater than 10 indicates that the IV SNPs are highly effective in minimizing potential bias (27), while a value of F less than or equal to 10 suggests that the SNPs are weak IVs.

### MR analysis

We conducted five MR techniques to assess the impact of lipoproteins on ED outcomes. The main MR approach used was the inverse variance weighted (IVW) method, while MR Egger, weighted median, simple mode, and weighted mode techniques were also employed as alternative options (28, 29). In our study, we utilized MVMR to assess the independent causal effects of lipoprotein characteristics on the occurrence of ED. The results of the MR analyses were reported as odds ratios (ORs) with corresponding 95% confidence intervals (CIs). The MR Egger and MR Pleiotropy RESidualSum and Outlier (MR‐PRESSO) method was used to assess the multiple effects. A p-value exceeding 0.05 indicated the absence of any multiplicity of effects. The Cochran’s Q statistic was examined using mr_egger and IVW, and a p-value greater than 0.05 indicated no heterogeneity. Furthermore, a ‘leave‐one‐out’ sensitivity analysis was performed to demonstrate that the impact of lipoproteins on ED outcomes remained unchanged by individual SNPs.

### Study subjects and exclusive criteria

The clinical data for this study were collected retrospectively from the medical records of 224 male patients who underwent physical examinations at the urology clinic from March 2019 to August 2022. Selected cases were required to have had persistent ED or suspected ED for more than 6 months with regular heterosexual relations (at least once per week) with a partner.

Exclusion criteria were advanced age (≥ 60 years), the presence of sleep disturbances, supportive medication prescriptions that may affect erectile function, pelvic trauma, spinal cord injuries, known history of atherosclerotic coronary heart disease, thyroid diseases, penile deformities, old or simultaneous neurologic diseases, hypogonadism, and other hormonal disorders. Patients who refused to respond to surveys and examinations were also excluded. A total of 212 patients who met the inclusion and exclusion criteria were included in the study. Prior to obtaining written permission, patients were provided with information about the purpose of the study, potential benefits, and potential hazards.

### Baseline data collection

All participants were required to complete a detailed data collection form. The form included questions about their age, height, weight, marital status, fertility status, family history of diabetes, hypertension, and coronary artery disease. Additionally, participants were asked about their drinking and smoking habits. The form also included assessments such as the Generalized Anxiety Disorder (GAD-7) score, the Patient Health Questionnaire-9 (PHQ-9) score, and IIEF-5 score (30). The GAD-7 was used to evaluate symptoms of anxiety, while the PHQ-9 was used to determine symptoms of depression (31, 32). A comprehensive physical examination was conducted on all patients, encompassing measurements of weight, height, body mass index (BMI), blood pressure, diabetes, hypertension, and the status of coronary artery disease. Additionally, thyroid hormones, sex hormones, and lipid profiles were assessed, including serum total glucose (GLU), thyroid-stimulating hormone (TSH), estradiol (E), progesterone (P), luteinizing hormone (LH), prolactin (PRL), testosterone (T), follicle stimulating hormone (FSH), Apo B, Apo A1, lipoprotein A, LDL, TG, HDL, TC, gamma glutamyl transferase (GGT), and lactate dehydrogenase (LDH).

### NPTR monitoring

The NPTR assessment was conducted on all participants using RigiScan monitoring. According to the study “Forensic identification of male sexual dysfunction” (GB/T37237-2018) conducted in China, effective penile erection criteria include RigiScan tests showing an average hardness of both the head and the root of the penis at maximum erection as greater than or equal to 60%, with a duration of more than or equal to 10 minutes (33). A multi-center study in Chinese men revealed that normal NPTR monitoring during 8 hours of sleep at night showed more than 2 effective erections of the penis, each lasting more than 10 minutes, with an erection hardness exceeding 60% of the normal erection (34). Based on the criteria adopted in this study, more than 2 effective erections lasting more than 10 minutes were required, with erection hardness values exceeding 60%, and satisfaction of both the tip and base of the penis at least once each. Prior to going to sleep, participants were instructed to ensure restful sleep by avoiding napping, alcohol and caffeine intake, and emptying the bladder and bowel. Based on the results of NPTR monitoring, patients were categorized into organic ED and psychogenic ED groups.

### Statistical analysis

The R 4.3.4 software (R Foundation for Statistical Computing, Vienna, Austria) was utilized for conducting MR analyses in this study. At various points, packages such as ‘TwoSampleMR’, ‘MR-PRESSO’, ‘forestplot’, and ‘MendelianRandomization’ were employed. Statistical significance of causality was attributed to SVMR and MVMR analyses, with a p-value of less than 0.05.

In the case-control study, statistical analysis of all case data was performed using SPSS Statistics (version 26.0; SPSS for Windows). The one-sided Kolmogorov-Smirnov test was used to examine the presence of a normal distribution. The student’s t-test was employed for the purpose of comparing continuous variables with a normal distribution, which were presented as the average ± standard deviation (SD). The chi square test or Fisher’s exact chi-square test was used for categorical variables. For quantitative data that were not normally distributed, the median (quartile) was reported, and the non-parametric tests were used for comparison. Receiver operating characteristic (ROC) curve analysis was also performed to ensure the cut-off values for the relative variables. Additionally, binary logistic regression analyzes were carried out to determine the ORs and accompanying 95% CIs for potential effective variables, such as Apo B, Apo A1, and Apo A1/Apo B, in the prediction of organic vs psychogenic ED. A threshold of *p* <.05 (two-tailed) was used to determine statistical significance.

## Results

### Single variable MR

By performing a series of IVs selection steps, we identified the following IVs: 48 SNPs as Apo A1, 36 SNPs as Apo B, 52 SNPs as Apo B/A1, 277 SNPs as HDL, 146 SNPs as LDL, 67 SNPs as high cholesterol, and 224 SNPs as TG. These selection steps included criteria such as p<5×10^-8^, r^2^<0.001 and kb>10,000, non-palindromic, F-value > 10. Additionally, we excluded SNPs associated with fasting glucose, uric acid, and hypertension using the PhenoScannerV2 database (**Figure 1**). The p-value of the fixed effect IVW for all seven lipoproteins was greater than 0.05, indicating that there is no causal relationship between ED formation and the results of the MR-Egger regression. Furthermore, the Cochrane Q test, MR-PRESSO, and ‘leave-one-out’ sensitivity analyses demonstrated that the data was not significantly heterogeneous or pleiotropic (**Supplementary Figures 1**, **2**). Given the robust interactions observed among the seven lipoproteins, additional MVMR analysis was conducted to evaluate their individual effects.

### Multivariate MR

**Figure 2:**
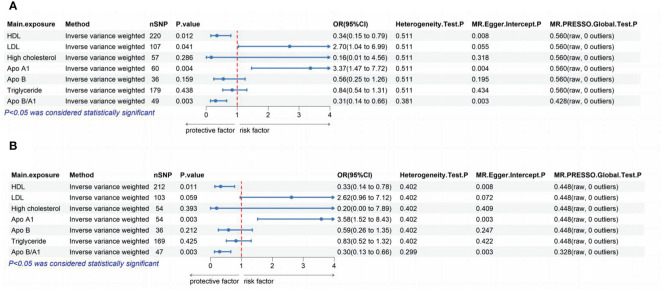
The lipoproteins were analyzed to determine their independent effects using MVMR. **(A)**: The independent effects of each of the 7 lipoproteins were assessed without excluding confounders such as fasting glucose, uric acid, and hypertension. **(B)**: The independent effects of each of the 7 lipoproteins were analyzed after excluding confounders such as fasting glucose, uric acid, and hypertension. MR, Mendelian Randomization; MVMR, multivariate MR; SNP, single nucleotide polymorphism; HDL, high-density lipoprotein; LDL, low-density lipoprotein; Apo, apolipoprotein; OR, odds ratio; CI, confidence interval; MR‐PRESSO, MR Pleiotropy RESidualSum and Outlier.

The outcomes of the MVMR analysis are presented in **Figure 2**. To account for the covariance of Apo A1 and Apo B with Apo B/A1, we divided them into two groups for the MVMR analysis. When considering confounders such as fasting glucose, uric acid, and hypertension, HDL, LDL, Apo A1, and Apo B/A1 were all found to have a causal association with the development of ED. The corresponding p-values were 0.012, 0.041, 0.004, and 0.003, respectively. The OR and 95% CI were 0.34 (0.15-0.79), 2.7 (1.04-6.99), 3.37 (1.47-7.72), and 0.31 (0.14-0.66). After excluding these confounders, HDL, Apo A1, and Apo B/A1 remained causally associated with the development of ED. The p-values were 0.011, 0.003, and 0.003, with corresponding OR and 95% CI of 0.33 (0.14-0.78), 3.58 (1.52-8.43), and 0.30 (0.13-0.66), respectively. Sensitivity analyses using MR-Egger regression, Cochrane Q test, and MR-PRESSO indicated no significant heterogeneity or pleiotropy in the data.

### Case-control study

**Figure 3:**
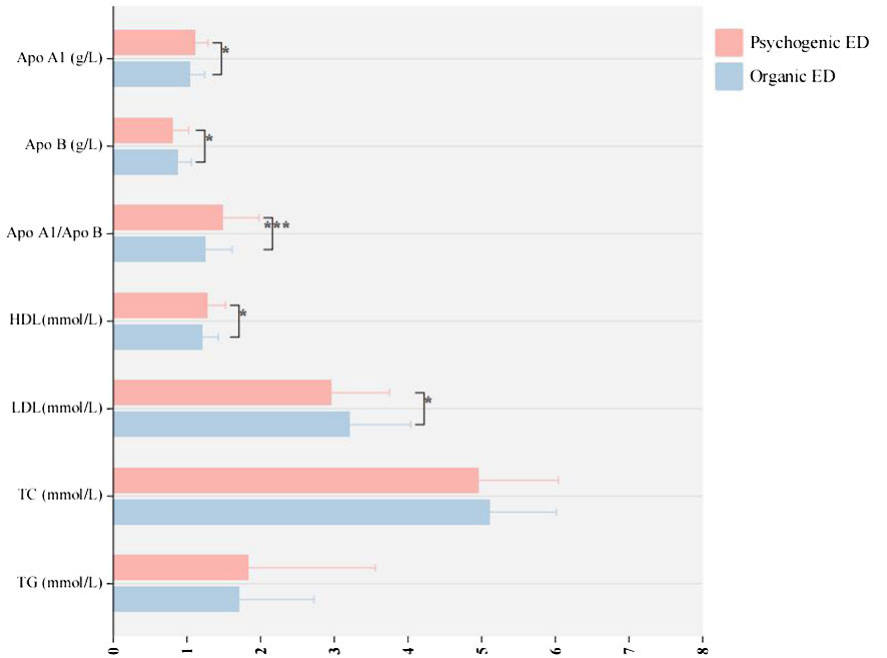
The study examines the variations in lipoprotein levels between organic and psychogenic ED. ED, erectile dysfunction; HDL, high-density lipoprotein; LDL, low-density lipoprotein; TG, triglyceride; TC, total cholesterol; Apo, apolipoprotein. *p < 0.05; ***p < 0.005.

The research included a total of 212 patients. **Table 2** presents the main social, biochemical, and clinical characteristics of all the patients. The indicators for all patients were analyzed for correlation, and the results are shown in **Supplementary Figure 3**. **Table 3** provides information on the main social demographic, biochemical and clinical characteristics of patients in the normal NPTR (psychogenic ED) and abnormal NPTR (organic ED) outcome groups. Based on the NPTR results, 90 participants had psychogenic ED, while 122 had organic ED. Our investigation revealed no significant differences in age or SP between the two groups (32.51 ± 6.81 vs. 33.02 ± 7.35, 122.20 ± 12.29 vs. 120.80 ± 12.02, respectively). Compared to the organic ED group, the psychogenic ED group had significantly higher IIEF-5 scores (*P = .*019). Although not statistically significant, the psychogenic ED group had higher GAD-7 and PHQ-9 scores compared to the group of organic ED (*P = .*649 and *P = .*593). Furthermore, E2 levels were significantly decreased in the organic ED group (*P = .*045). Regarding the lipid profile, **Figure 3** demonstrates that the average levels of Apo B and LDL were considerably higher in the organic ED group compared to the psychogenic ED group (*P* = .024 and *P* = .029). It is worth mentioning that there was a significant decrease in the levels of Apo A1, Apo A1/B, and HDL (*P* = .019, P <.001, and *P* = .027). However, there were no significant differences in TC or TG levels between the two groups (*P* = .265 and *P* = .548).

**Table 2:** General characteristics of all patients.

Characteristics	All patients (n = 212)
Age (years)	32.81 ± 7.11
BMI (kg/m^2^)	24.97 ± 3.57
SP (mmHg)	121.40 ± 12.13
Hypertension	14 (6.60%)
Diabetes	10 (4.72%)
Cigarette smoking	86 (40.57%)
Alcohol consumption	93 (43.87%)
Apolipoprotein B (g/L)	0.84 ± 0.20
Apolipoprotein A1 (g/L)	1.07 ± 0.19
Lipoprotein A (mg/L)	88.70 (42.90, 186.80)
TC (mmol/L)	5.05 ± 0.98
TG (mmol/L)	1.42 (0.96, 2.05)
HDL (mmol/L)	1.23 ± 0.24
LDL (mmol/L)	3.10 ± 0.82
GGT (U/L)	26.00 (18.00, 42.75)
LDH (U/L)	166.50 (146.93, 183.08)
GLU (mmol/L)	5.05 (4.80, 5.40)
FSH (IU/L)	3.46 (2.47, 4.76)
LH (IU/L)	3.04 (2.24, 3.90)
PRL (ng/mL)	13.68 (9.45, 18.81)
E2 (pg/mL)	25.11 ± 9.42
P (ng/mL)	0.20 (0.13, 0.27)
T (ng/dL)	501.06 ± 193.02
TSH (uIU/mL)	2.23 ± 1.20
IIEF-5	12.95 ± 4.72
GAD-7	6.00 (3.00, 11.00)
PHQ-9	5.00 (3.00, 9.00)

Normally distributed data are expressed as mean ± standard deviation, and non-normally distributed data are expressed as median (quartile).

Apo, apolipoprotein; BMI, Body Mass Index; E2, estradiol; FSH, follicle stimulating hormone; GAD-7, the Generalized Anxiety Disorder; GGT, gamma glutamyl transferase; GLU, serum total glucose; HDL, high-density lipoprotein; IIEF-5, the International Index of Erectile Function; LDH, lactate dehydrogenase; LDL, low-density lipoprotein; LH, luteinizing hormone; P, progesterone; PHQ-9, the Patient Health Questionnaire-9; SP, systolic blood pressure; T, testosterone; TC, total cholesterol; TG, triglyceride; TSH, thyroid stimulating hormone.

**Table 3:** Associations between the baseline data and ED.

Characteristics	Psychogenic ED (n = 90)	Organic ED (n = 122)	*P*
Age (years)	32.51 ± 6.81	33.02 ± 7.35	.604
BMI (kg/m^2^)	24.76 ± 3.71	25.12 ± 3.47	.465
SP (mmHg)	122.20 ± 12.29	120.80 ± 12.02	.408
Lipoprotein A (mg/L)	88.30 (44.40, 190.55)	89.70 (41.48, 176.48)	.731
GGT (U/L)	26.00 (17.75, 48.00)	26.00 (19.00, 39.00)	.883
LDH (U/L)	172.60 (149.75, 188.25)	164.00 (145.75, 181.25)	.179
GLU (mmol/L)	5.29 ± 1.19	5.42 ± 1.49	.508
FSH (IU/L)	3.60 (2.41, 5.23)	3.41 (2.48, 4.26)	.289
LH (IU/L)	3.13 (2.16, 4.18)	3.02 (2.28, 3.84)	.555
PRL (ng/mL)	14.27 (10.52, 19.79)	13.13 (8.67, 18.68)	.085
E2 (pg/mL)	26.62 ± 10.65	23.96 ± 8.23	.045*
P (ng/mL)	0.21 (0.13, 0.27)	0.19 (0.12, 0.27)	.507
T (ng/dL)	513.96 ± 194.24	491.28 ± 192.36	.420
TSH (uIU/mL)	2.19 ± 1.32	2.25 ± 1.10	.715
IIEF-5	13.83 ± 4.63	12.30 ± 4.70	.019*
GAD-7	7.00 (3.00, 12.00)	6.00 (3.00, 10.00)	.649
PHQ-9	5.50 (3.00, 9.25)	5.00 (3.00, 9.00)	.593

Normally distributed data are expressed as mean ± standard deviation and analyzed using the t-test. non-normally distributed data are expressed as median (quartile) and analyzed using Rank sum test. *P <.05.

BMI, Body Mass Index; E2, estradiol; FSH, follicle stimulating hormone; GAD-7, the Generalized Anxiety Disorder; GGT, gamma glutamyl transferase; GLU, serum total glucose; IIEF-5, the International Index of Erectile Function; LDH, lactate dehydrogenase; LH, luteinizing hormone; P, progesterone; PHQ-9, the Patient Health Questionnaire-9; SP, systolic blood pressure; T, testosterone; TSH, thyroid stimulating hormone.

In our study, we divided the 8 lipid profile objectives into grades (Q1, Q2, Q3, and Q4) using four quartiles. We then analyzed the proportion of individuals with organic ED and psychogenic ED based on different lipid profile levels (**Table 4**). The results showed that patients with higher Apo A1 and Apo A1/Apo B ratios had a significantly lower proportion of organic ED compared to those with psychogenic ED (*P = .*018 and *P = .*001, respectively). Additionally, we performed binary logistic regression analysis (**Table 5**) to investigate the relationship between various lipid profiles and the prevalence of organic ED. Our findings revealed that the presence of organic ED was significantly influenced by Apo A1, Apo A1/B and HDL (*P = .*033, *P = .*001 and P=.007, respectively), while Apo B, lipoprotein A, TC, TG and LDL did not show significant associations (*P = .*091, *P = .*682, *P = .*695, *P = .*398 and P=.561, respectively) in our study.

**Table 4:** Comparison of the number of participants with organic ED and different lipid profiles.

Characteristics	Q1	Q2	Q3	Q4	P
Apolipoprotein B (g/L)	26 (48.1%)	33 (57.9%)	29 (58.0%)	34 (66.7%)	.296
Apolipoprotein A1 (g/L)	42 (75.0%)	25 (52.1%)	29 (54.7%)	26 (47.3%)	.018*
Apo A1/Apo B	38 (71.7%)	37 (69.8%)	25 (47.2%)	22 (43.5%)	.001**
Lipoprotein A (mg/L)	31 (59.6%)	29 (54.7%)	33 (62.3%)	29 (54.7%)	.821
TC (mmol/L)	29 (54.7%)	31 (58.5%)	34 (63.0%)	28 (53.8%)	.769
TG (mmol/L)	27 (49.1%)	32 (62.7%)	32 (60.4%)	31 (58.5%)	.500
HDL(mmol/L)	38 (71.7%)	31 (55.4%)	28 (52.8%)	25 (50.0%)	.103
LDL(mmol/L)	24 (45.3%)	30 (56.6%)	35 (64.8%)	33 (63.5%)	.163

Apo, apolipoprotein; TC, total cholesterol; TG, triglyceride; HDL, high-density lipoprotein; LDL, low-density lipoprotein. Statistical methods were analyzed using ANOVA. *P <.05; **P <.005.

**Table 5:** Based on binary logistic regression analyses, correlations between lipid profiles and occurrence of abnormal NPTR results.

Characteristics	B	OR (95% CI)	*P*
Apo B (g/L)	2.631	13.888 (0.655–294.574)	.091
Apo A1 (g/L)	-1.922	0.146 (0.025–0.855)	.033*
Apo A1/Apo B	-1.474	0.229 (0.095–0.549)	.001**
Lipoprotein A (mg/L)	0.000	1 (0.998–1.001)	.682
TC (mmol/L)	-0.135	0.874 (0.446–1.714)	.695
TG (mmol/L)	-0.113	0.893 (0.688–1.161)	.398
HDL (mmol/L)	-2.199	0.111(0.022-0.553)	.007**
LDL (mmol/L)	.225	1.253(.586-2.679)	.561

Apo, apolipoprotein; TC, total cholesterol; TG, triglyceride; HDL, high-density lipoprotein; LDL, low-density lipoprotein; OR, odds ratio; CI, confidence interval. *P <.05; **P <.005.

**Figure 4:**
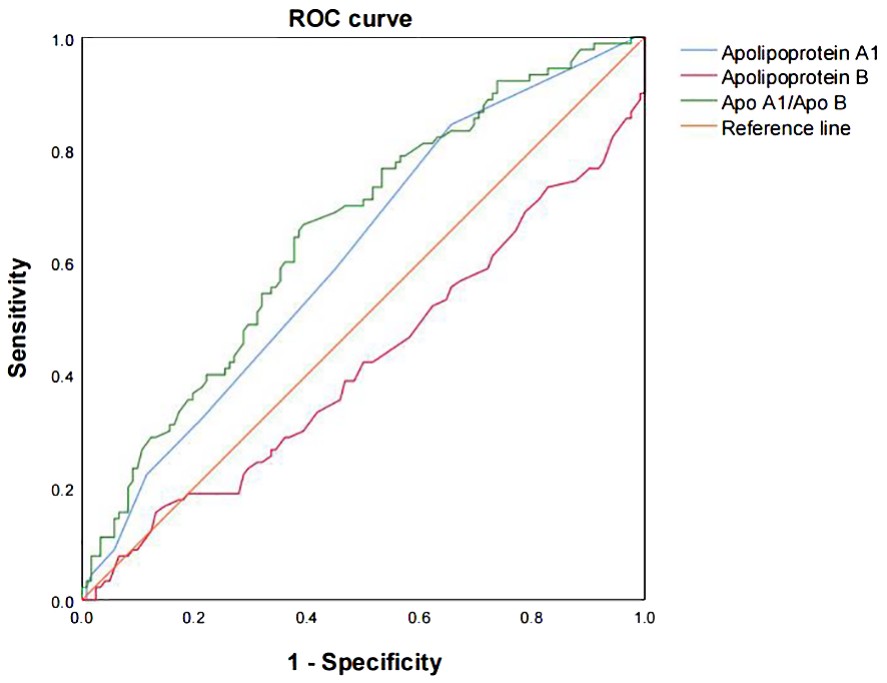
The ROC curves were analyzed to evaluate the prediction scores of serum Apo A1 and Apo A1/B in relation to the different NPTR scores. The ROC curve was used to diagnose organic ED based on the various NPTR results. The AUC values for Apo A1, Apo B, and Apo A1/B were 0.61 (95% CI: 0.54–0.69, *P* = .005, cut-off < 0.95), 0.42 (95% CI: 0.34–0.50, *P* = .051, cut-off < 1.09), and 0.66 (95% CI: 0.58–0.73, *P* <.001, cut-off < 1.26), respectively. Apo, apolipoprotein; AUC, the area under the curve; CI, confidence interval; ROC, receiver operating characteristic.

Apo A1 and Apo A1/B were analyzed using ROC curves to differentiate between organic and psychogenic ED (**Figure 4**). The diagnosis of organic ED was investigated based on the NPTR results. Out of the 8 markers, only two (Apo A1 and Apo A1/B) showed a moderate diagnostic value for organic ED, as indicated by the ROC curves (the area under the curve (AUC) = 0.61, 95% CI: 0.54–0.69, *P = .*005, cut-off < 0.95; AUC = 0.66, 95% CI: 0.58–0.73, *P <*.001, cut-off < 1.26, respectively).

## Discussion

ED is a multifaceted condition involving various factors like vascular, neurogenic, hormonal, psychogenic, vascular, and structural cave variables (35). Recent research has indicated that ED may be a potential precursor to CVD. Studies conducted in a clinical setting have demonstrated a powerful link between the emergence of ED and cardiovascular risk factors such as age, smoking, hypertension, obesity, diabetes, and dyslipidemia (36).

Our MR study did not find any significant causality in the SVMR of the seven lipoproteins, which aligns with previous MR studies (37). The probable explanation for this lack of causality is the intricate interplay among these lipoproteins (38), which cannot be disregarded solely based on SVMR. We further analyzed their respective independent effects using MVMR, and found that HDL, LDL, Apo A1, and Apo B/A1 were causally associated with the development of ED. However, after accounting for confounding factors such as fasting glucose, uric acid, and hypertension, the causal relationship between LDL and ED occurrence was no longer significant. This suggests that the influence of diabetes, hyperuricemia, and hypertension on ED occurrence may be mediated through their effects on LDL.

Our case-control study found no significant relationship between age and the prevalence of organic ED. This observation may be attributed to the fact that the patients attending Chinese andrology clinics were predominantly young. It is worth noting that the mean IIEF-5 score was lower in the organic ED group. Although the GAD-7 and PHQ-9 scores were higher in the psychogenic ED group compared to the organic ED group, this difference was not statistically significant. In contrast to a previous study, our findings indicate that NPTR monitoring is effective in distinguishing between organic and psychogenic ED (39).

Several risk factors are common to both ED and CVD (40, 41). It is important to recognize the connection between ED and CVD in order to effectively prevent and treat both conditions (42, 43). Additionally, ED significantly impacts patients’ quality of life. Therefore, it is crucial to examine the functions of Apo B, Apo A1, and Apo A1/Apo B in diagnosing organic ED, as these lipid profile markers have diagnostic significance in CVD (44). By doing so, medical professionals in various departments, even those unfamiliar with andrology, will be better equipped to treat and prevent CVD and ED in patients.

In the liver, Apo A1 is produced, an individual glycoprotein that makes up lipoprotein A, which is covalently linked to the lipoprotein Apo B-100 (45). The properties of Apo A1 can be influenced by various factors, including its size, sequence polymorphism, the type of lipoproteins it binds to, and the level of inflammation in the arterial wall. These factors suggest that Apo A1 may play a role in preventing atherosclerosis (46). On the other hand, Apo B, which is produced through cleavage and degradation of low density lipoproteins, contributes to the development of atherosclerosis (47). Apo B leads to vascular dysfunction, deposition of lipids and blood vessels in the walls of blood vessels, and the formation of foam cells (48). Considering this, it is possible that Apo A1’s ability to restore the artery wall could potentially prevent the development of organic ED in individuals.

A lower Apo A1/B ratio may indicate a higher risk of CVD (49). The ratio of HDL/LDL, represented by Apo A1/B, is inversely and favorably correlated with the probability of vascular damage (50). In our research, we found that Apo A1 and Apo A1/B were significantly lower in the organic ED compared to psychogenic ED. Apo A1 and Apo A1/B showed moderate diagnostic values for distinguishing between organic and psychogenic ED, according to the ROC curves. Among these results, Apo A1/B had the best diagnostic value for predicting organic ED. We also validated these findings using binary logistic regression analysis.

It is noteworthy that MVMR findings suggest link between HDL, Apo A1, and Apo B/A1 and the emergence of ED (OR and 95% CI 0.33 (0.14-0.78), 3.58 (1.52-8.43) and 0.30 (0.13-0.66), respectively). However, in the case-control study, binary logistic regression analyses indicate a potential association between HDL, Apo A1 and Apo A1/B and the occurrence of ED (OR and 95% CI of 0.11 (0.02-0.55), 0.15 (0.03-0.90) and 0.23 (0.10-0.55)). These findings support a causal relationship between HDL, Apo A1, and Apo A1/B and the development of ED. However, a paradox exists regarding the role of Apo A1 and Apo A1/B. This could be attributed to the diagnostic criteria used in MR, which do not clearly differentiate between organic and psychogenic ED. It is unlikely that there is a necessary connection between psychogenic ED and Apos. This highlights the need for a more definitive diagnosis of ED, considering its diagnostic aspects, to improve treatment and prognosis. Additionally, further research is required to understand the specific mechanism of action of Apo A1 in ED.

The mass spectra of other lipids, such as lipoprotein A, TC, and TG levels, did not provide any valuable information for diagnosing organic ED. Prior studies have demonstrated a link between elevated lipoprotein levels and a significantly increased risk of cardiovascular events in individuals with CVD (51, 52). However, despite higher levels of lipoprotein A in patients with organic ED, no significant correlation was observed. This could be attributed to the relatively young age of the participants and the exclusion of patients with CVD in our study.

Our research revealed that organic ED patients exhibited significantly lower levels of E2. The role of E2 in male sexual function remains unidentified, and studies investigating the impact of estrogens on erectile function have yielded inconsistent findings (53–55). Notably, our study findings differed significantly from previous research. This discrepancy may be attributed to the fact that the control group, comprising individuals with psychogenic ED, did not represent a healthy population, as evidenced by high levels of anxiety and depression indicated in the questionnaires. Consequently, these factors may have contributed to the reported increase in E2 levels (56).

It is important to note that this study has certain limitations. To begin with, MR analysis places a great emphasis on pleiotropy. The existence of pleiotropy may violate the basic MR assumptions and further distort the findings. We applied two methods MR-Egger and MR-PRESSO to detect the horizontal pleiotropy. Although sensitivity analyses did not reveal clear pleiotropy, we cannot completely exclude the possible pleiotropy of genetic variants. Additionally, the case-control study lacked a normal healthy population as a control group and had a limited sample size, with data collected from only one center. Despite having a larger sample size compared to similar studies, it is important to interpret the results with caution. Lastly, we did not conduct concurrent sleep monitoring to directly observe the sleep of the patients. However, we did provide patients with instructions on how to ensure a good night’s sleep prior to the study.

This research has certain advantages. At present, there is a scarcity of studies investigating the correlation between Apos and ED. This research, involving both European and Chinese participants, was created to supplement this area of study. Moreover, the MR design is advantageous as it permits the derivation of causal inference rather than a correlation. The MR analyses took into account a thorough IV selection and multiple sensitivity analyses, guaranteeing adherence to three fundamental assumptions in MR. Furthermore, in case-control studies, unlike previous studies that relied heavily on the subjective IIEF-5 score, our study utilized NPTR monitoring results to distinguish between organic and psychogenic ED. As a result, we confirmed that Apos are linked to organic ED, but not to psychogenic ED.

## Conclusion

The MR study and case-control study have both validated the indispensable contribution of HDL, ApoA1, and ApoA1/B in the progression of ED. The differential diagnosis of ED between organic and psychogenic by NPTR testing further suggests that organic ED is characterized by reduced serum Apo A1 and Apo A1/B levels, as well as elevated Apo B levels. On the other hand, psychogenic ED does not exhibit these same associations. These findings suggest that Apo A1 and Apo A1/Apo B, which serve as serum indicators for organic ED risk, are valuable and play a critical role in the etiology of ED. Monitoring the levels of Apo A1 and Apo A1/B in the general population shows promise as a technique for identifying individuals with organic ED.

## Data availability statement

Publicly available datasets were analyzed in this study. This data can be found here: https://r5.finngen.fi/, http://www.ukbiobank.ac.uk, https://gwas.mrcieu.ac.uk/. Clinical data are available from the corresponding authors upon reasonable request.

## Ethics statement

The studies involving humans were approved by Tianjin Medical University General Hospital, Reg. No.ZYY-IRB-ZD-004(F)-002-02. The studies were conducted in accordance with the local legislation and institutional requirements. The participants provided their written informed consent to participate in this study. All subjects in the original genome-wide association studies provided informed consent. The original studies provide access to ethical review and approval for GWAS datasets.

## Author contributions

ZZ: Conceptualization, Data curation, Software, Writing – original draft, Writing – review & editing. MY: Conceptualization, Software, Writing – original draft. YL: Data curation, Software, Writing – original draft. YP: Formal analysis, Software, Writing – original draft. SW: Data curation, Methodology, Software, Writing – original draft. MX: Formal analysis, Visualization, Writing – original draft. HZ: Formal analysis, Visualization, Writing – original draft. XL: Supervision, Writing – review & editing, Conceptualization.

## Supplementary material

Supplementary Figure 1 Examining the causal association between lipoprotein traits and ED traits using SVMR. MR: Mendelian randomization; SNP: single nucleotide polymorphism; HDL: high-density lipoprotein; LDL: low-density lipoprotein; Apo: apolipoprotein; OR: odds ratio; CI: confidence interval; MR‐PRESSO: MR Pleiotropy RESidualSum and Outlier.

Supplementary Figure 2 “Leave-one-out” sensitivity analysis was **conducted for lipoproteins.** The sensitivity analyses included: **(A)** Apo A1, **(B)** Apo B, **(C)** Apo B/A1, **(D)** HDL, **(E)** LDL, **(F)** high cholesterol, and **(G)** TG. MR: Mendelian randomization; HDL: high-density lipoprotein; LDL: low-density lipoprotein; Apo: apolipoprotein; TG: triglyceride.

Supplementary Figure 3 Correlation analysis was conducted on collected indicators for all patients. Apo: apolipoprotein; BMI: Body Mass Index; E2: estradiol; FSH: follicle stimulating hormone; GAD-7: Generalized Anxiety Disorder; GGT: gamma glutamyl transferase; GLU: serum total glucose; HDL: high-density lipoprotein; IIEF-5: the International Index of Erectile Function; LDH: lactate dehydrogenase; LDL: low-density lipoprotein; LH: luteinizing hormone; P: progesterone; PHQ-9: the Patient Health Questionnaire-9; SP: systolic blood pressure; T: testosterone; TC: total cholesterol; TG: triglyceride. **P* <.05; ***P* <.005; ****P* <.001.
