# Supplementary material for: The indicative effects of apolipoproteins on organic erectile dysfunction: bridging Mendelian randomization and case-control study
Source: Front Endocrinol (Lausanne). 2024 Jun 13;15:1359015. doi: 10.3389/fendo.2024.1359015 (PMC11208309; doi:10.3389/fendo.2024.1359015)
Supplement: Supplementary Figure 1 — Examining the causal association between lipoprotein traits and ED traits using SVMR. MR: Mendelian randomization; SNP: single nucleotide polymorphism; HDL: high-density lipoprotein; LDL: low-density lipoprotein; Apo: apolipoprotein; OR: odds ratio; CI: confidence interval; MR‐PRESSO: MR Pleiotropy RESidualSum and Outlier. [file DataSheet_1.zip › Supplementary Figure 1.pdf]

Main.exposure	Method	nSNP	P.value	OR(95%CI)	Heterogeneity.Test.P	MR.Egger.Intercept.P	MR.PRESSO.Global.Test.P
apo A1	MR Egger	48	0.318	0.78(0.49 to 1.26)	0.344		
	Weighted median	48	0.501	0.87(0.57 to 1.31)			
	Inverse variance weighted	48	0.814	0.97(0.73 to 1.29)	0.335	0.282	0.374(raw, 0 outliers)
	Simple mode	48	0.823	0.92(0.43 to 1.96)			
	Weighted mode	48	0.995	1.00(0.66 to 1.53)			
apo B	MR Egger	36	0.776	0.92(0.53 to 1.61)	0.116		
	Weighted median	36	0.970	0.99(0.63 to 1.55)			
	Inverse variance weighted	36	0.837	0.97(0.70 to 1.34)	0.139	0.838	0.133(raw, 0 outliers)
	Simple mode	36	0.852	0.91(0.34 to 2.46)			
	Weighted mode	36	0.666	1.13(0.65 to 1.98)			
apo B/A1	MR Egger	52	0.868	0.95(0.56 to 1.64)	0.096		
	Weighted median	52	0.891	0.97(0.61 to 1.53)			
	Inverse variance weighted	52	0.336	0.86(0.63 to 1.17)	0.109	0.636	0.099(raw, 0 outliers)
	Simple mode	52	0.081	0.43(0.17 to 1.09)			
	Weighted mode	52	0.759	1.09(0.63 to 1.88)			
HDL	MR Egger	277	0.699	1.07(0.75 to 1.53)	0.236		
	Weighted median	277	0.088	1.38(0.95 to 2.00)			
	Inverse variance weighted	277	0.311	1.12(0.90 to 1.41)	0.247	0.743	0.258(raw, 0 outliers)
	Simple mode	277	0.742	1.13(0.55 to 2.32)			
	Weighted mode	277	0.156	1.36(0.89 to 2.07)			
LDL	MR Egger	146	0.233	0.74(0.45 to 1.21)	0.232		
	Weighted median	146	0.324	0.80(0.51 to 1.25)			
	Inverse variance weighted	146	0.875	0.98(0.72 to 1.32)	0.214	0.163	0.248(raw, 0 outliers)
	Simple mode	146	0.726	0.87(0.39 to 1.94)			
	Weighted mode	146	0.333	0.80(0.50 to 1.26)			
High cholesterol	MR Egger	67	0.836	0.71(0.03 to 16.83)	0.154		
	Weighted median	67	0.667	0.57(0.05 to 7.20)			
	Inverse variance weighted	67	0.883	0.88(0.15 to 5.01)	0.175	0.879	0.168(raw, 0 outliers)
	Simple mode	67	0.540	3.95(0.05 to 312.52)			
	Weighted mode	67	0.618	2.08(0.12 to 36.92)			
triglyceride	MR Egger	224	0.567	1.10(0.79 to 1.53)	0.249		
	Weighted median	224	0.782	0.95(0.64 to 1.39)			
	Inverse variance weighted	224	0.450	1.09(0.87 to 1.37)	0.264	0.942	0.253(raw, 0 outliers)
	Simple mode	224	0.298	0.67(0.32 to 1.42)			
	Weighted mode	224	0.462	0.88(0.62 to 1.24)			

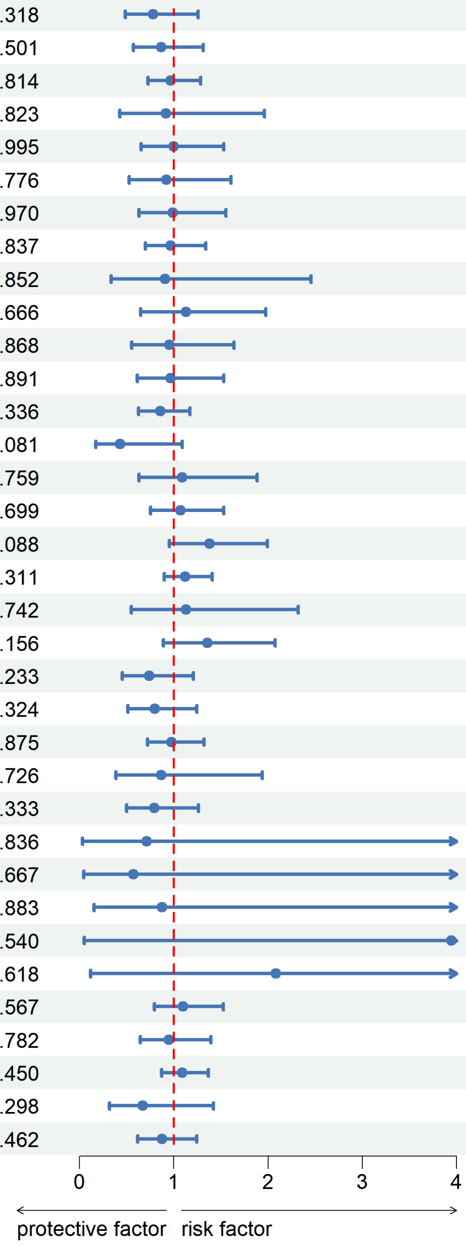

P<0.05 was considered statistically significant
