# Supplementary figures and images for: The indicative effects of apolipoproteins on organic erectile dysfunction: bridging Mendelian randomization and case-control study
Source: Front Endocrinol (Lausanne). 2024 Jun 13;15:1359015. doi: 10.3389/fendo.2024.1359015 (PMC11208309; doi:10.3389/fendo.2024.1359015)

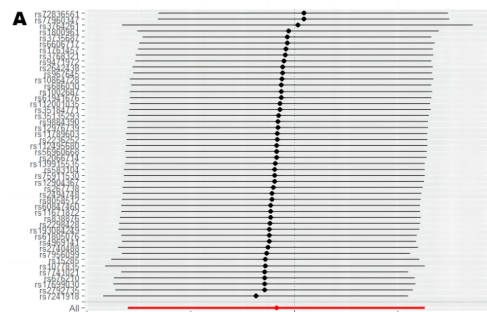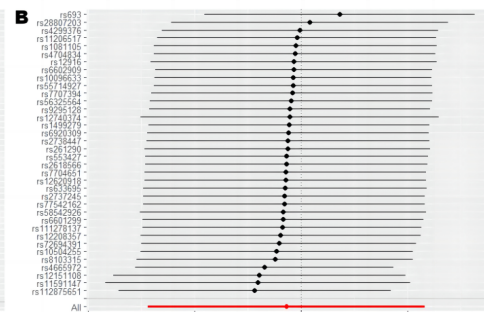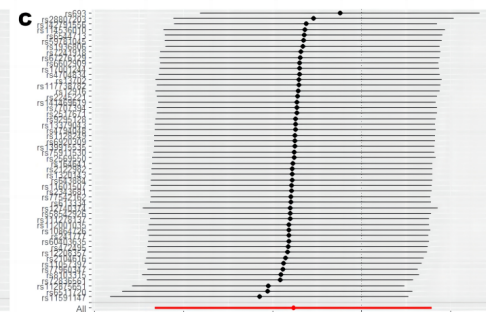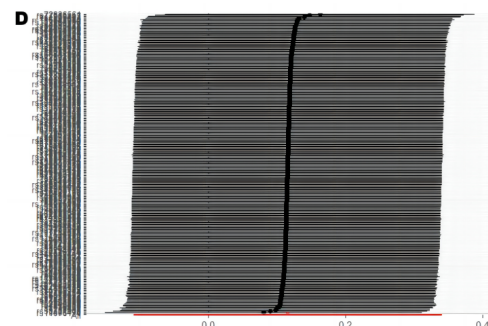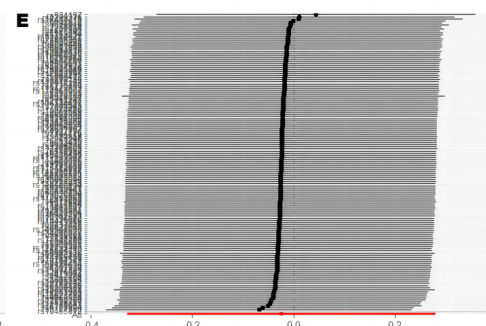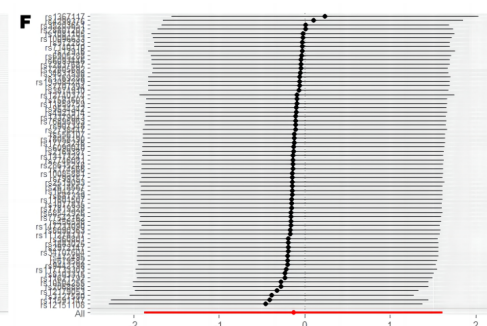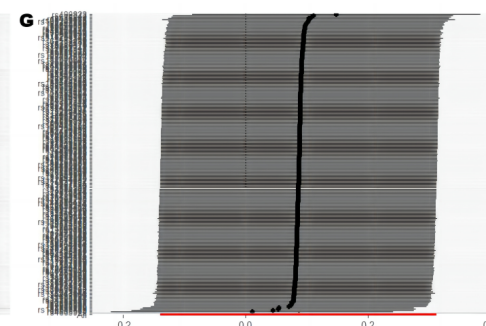

Supplement: Supplementary Figure 1 — Examining the causal association between lipoprotein traits and ED traits using SVMR. MR: Mendelian randomization; SNP: single nucleotide polymorphism; HDL: high-density lipoprotein; LDL: low-density lipoprotein; Apo: apolipoprotein; OR: odds ratio; CI: confidence interval; MR‐PRESSO: MR Pleiotropy RESidualSum and Outlier. [file DataSheet_1.zip › Supplementary Figure 2.pdf]

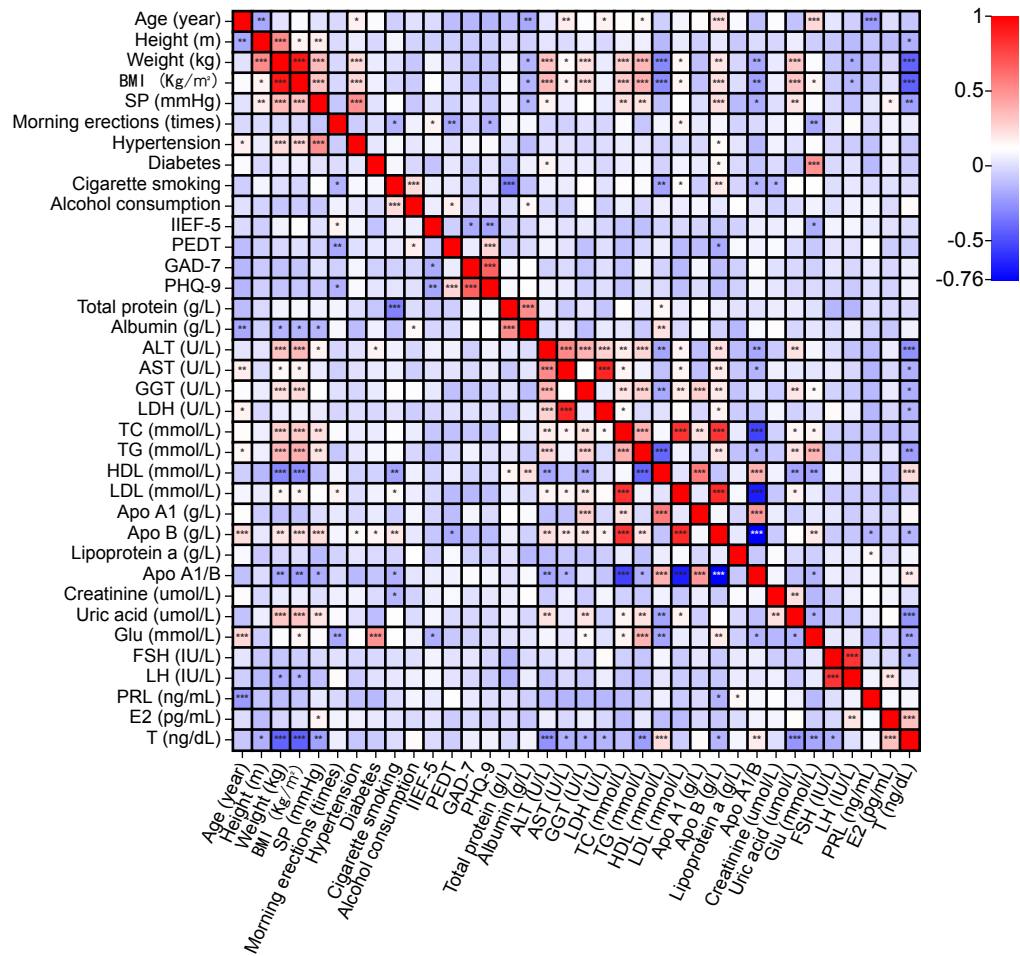

Supplement: Supplementary Figure 1 — Examining the causal association between lipoprotein traits and ED traits using SVMR. MR: Mendelian randomization; SNP: single nucleotide polymorphism; HDL: high-density lipoprotein; LDL: low-density lipoprotein; Apo: apolipoprotein; OR: odds ratio; CI: confidence interval; MR‐PRESSO: MR Pleiotropy RESidualSum and Outlier. [file DataSheet_1.zip › Supplementary Figure 3.pdf]
